# Broadening of Virus-Specific CD8^+^ T-Cell Responses Is Indicative of Residual Viral Replication in Aviremic SIV Controllers

**DOI:** 10.1371/journal.ppat.1005247

**Published:** 2015-11-04

**Authors:** Takushi Nomura, Hiroyuki Yamamoto, Hiroshi Ishii, Hirofumi Akari, Taeko K. Naruse, Akinori Kimura, Tetsuro Matano

**Affiliations:** 1 AIDS Research Center, National Institute of Infectious Diseases, Tokyo, Japan; 2 Center for Human Evolution Modeling Research, Primate Research Institute, Kyoto University, Inuyama, Aichi, Japan; 3 Department of Molecular Pathogenesis, Medical Research Institute, Tokyo Medical and Dental University, Tokyo, Japan; 4 The Institute of Medical Science, The University of Tokyo, Tokyo, Japan; Emory University, UNITED STATES

## Abstract

Control of HIV replication is a rare immunological event, providing clues to understand the viral control mechanism. CD8^+^ T-cell responses are crucial for virus control, but it is unclear whether lasting HIV containment can be achieved after establishment of infection. Here, we describe lasting SIV containment in a macaque AIDS model. Analysis of ten rhesus macaques that controlled viremia for 2 years post-infection found accumulation of proviral *gag* and *nef* CD8^+^ T-cell escape mutations in four of them. These four controllers mounted CD8^+^ T cells targeting Gag, Nef, and other viral proteins at 4 months, suggesting that broadening of CD8^+^ T-cell targets can be an indicator of the beginning of viral control failure. The remaining six aviremic SIV controllers, however, harbored proviruses without mutations and showed no or little broadening of their CD8^+^ T-cell responses in the chronic phase. Indeed, three of the latter six exhibiting no change in CD8^+^ T-cell targets showed gradual decreases in SIV-specific CD8^+^ T-cell frequencies, implying a concomitant reduction in viral replication. Thus, stability of the breadth of virus-specific CD8^+^ T-cell responses may represent a status of lasting HIV containment by CD8^+^ T cells.

## Author Summary

CD8^+^ T-cell responses are crucial for HIV control, but it is unclear whether lasting HIV containment can be achieved after establishment of infection. Several T cell-based vaccine trials have currently shown primary viremia control in macaque AIDS models of simian immunodeficiency virus (SIV) infection, but residual viral replication may occur, followed by accumulation of viral CD8^+^ T-cell escape mutations, possibly leading to eventual viremia rebound. In the present study, we analyzed ten rhesus macaques that controlled SIV replication without detectable viremia for more than 2 years. Animals were divided into two groups on the basis of proviral genome sequences at 2 years post-infection. Analysis of the first group exhibiting multiple CD8^+^ T-cell escape mutations indicated that broadening of CD8^+^ T-cell responses can be an indicator of the beginning of viral control failure. Conversely, analysis of the second group having no mutation suggested that stability of the breadth of virus-specific CD8^+^ T-cell responses represents a status of lasting HIV containment by CD8^+^ T cells. Thus, this study presents a model of stable SIV containment, contributing to elucidation of the requisites for lasting HIV control.

## Introduction

Human immunodeficiency virus (HIV) and simian immunodeficiency virus (SIV) infection induces chronic, persistent viral replication leading to AIDS onset in humans and rhesus macaques, respectively. While antiretroviral therapy (ART) has reduced the morbidity and mortality due to HIV, it does not cure infection. Much effort has been made aiming at inducing a functional cure, defined as HIV containment with cessation of ART [1–4]. A current trial of administration with a monoclonal broadly reactive neutralizing antibody under ART showed a longer aviremic period but eventual rebound viremia after ART interruption in rhesus macaques [5,6].

Virus-specific CD8^+^ T cells exert strong suppressive pressure on HIV/SIV replication [7–11], but fail to control viremia in most infections. Studies of HIV-infected individuals have revealed the association of certain HLA or major histocompatibility complex (MHC) class I genotypes with lower viral loads [12–15]. In the Indian rhesus macaque AIDS model, animals possessing protective MHC alleles such as Mamu-B*08 and Mamu-B*17 tend to show slower disease progression after SIVmac251/SIVmac239 infection [16–18]. CD8^+^ T-cell responses restricted by these HLA/MHC molecules have been shown to be responsible for HIV/SIV control in most studies [15,19–21]. However, aviremic HIV/SIV control is rare, and even in those with undetectable viremia, residual viral replication can occur and allow accumulation of viral genome mutations resulting in viral escape from CD8^+^ T-cell recognition, possibly leading to eventual viremia rebound [22–25].

Several prophylactic T cell-based vaccine trials have currently shown primary viremia control in macaque AIDS models [26–29]. However, it is difficult to obtain sterile protection from virus infection by T cell-based vaccines, and whether vaccine-based, primary non-sterile viral control can be stably maintained is debatable. Analysis of those rare cases exhibiting aviremic HIV/SIV control may provide clues to the development of a novel intervention resulting in lasting HIV control.

We previously developed a prophylactic AIDS vaccine using a DNA prime and a boost with a Sendai virus (SeV) vector expressing SIVmac239 Gag (SeV-Gag) [26,30]. Our trial showed vaccine-based control of an SIVmac239 challenge in a group of Burmese rhesus macaques sharing the MHC class I haplotype *90-120-Ia* (referred to as A^+^ animals) [31]. The Mamu-A1*043:*01*, Mamu-A1*065:*01*, Mamu-B*061:*03*, Mamu-B*068:*04*, and Mamu-B*089:*01* alleles have been confirmed in this haplotype [32,33]. Two-thirds of unvaccinated A^+^ animals showed persistent viremia after SIVmac239 infection, whereas all the A^+^ animals vaccinated with a DNA prime and an SeV-Gag boost controlled SIV replication without detectable viremia at 2 months post-challenge [31,33]. CD8^+^ T-cell responses specific for dominant Mamu-A1*043:01 (GenBank accession number AB444869)-restricted Gag_206–216_ (IINEEAADWDL) and Mamu-A1*065:01 (AB444921)-restricted Gag_241–249_ (SSVDEQIQW) epitopes are responsible for this vaccine-based SIV control [31]. However, two of these SIV controllers accumulated multiple *gag* CD8^+^ T-cell escape mutations and plasma viremia reappeared after 1 year of SIV control [25].

In the present study, we analyzed ten A^+^ animals that controlled viremia for 2 years after SIVmac239 challenge to determine whether such non-sterile aviremic SIV control can be stably maintained. Four of these ten SIV controllers exhibited an accumulation of proviral CD8^+^ T-cell escape mutations, indicating the latent viral control failure with preceding broadening of SIV-specific CD8^+^ T-cell targets. In contrast, the remaining six controllers showed no proviral mutations with little change in CD8^+^ T-cell targets in the chronic phase. Three of the latter six showed a gradual reduction in SIV-specific CD8^+^ T-cell frequencies, implying stable non-sterile SIV control with waning viral replication. These results suggest a possible achievement of lasting SIV containment by CD8^+^ T cells without transition of their targets.

## Results

### Proviral sequences in SIV controllers

**Fig 1:**
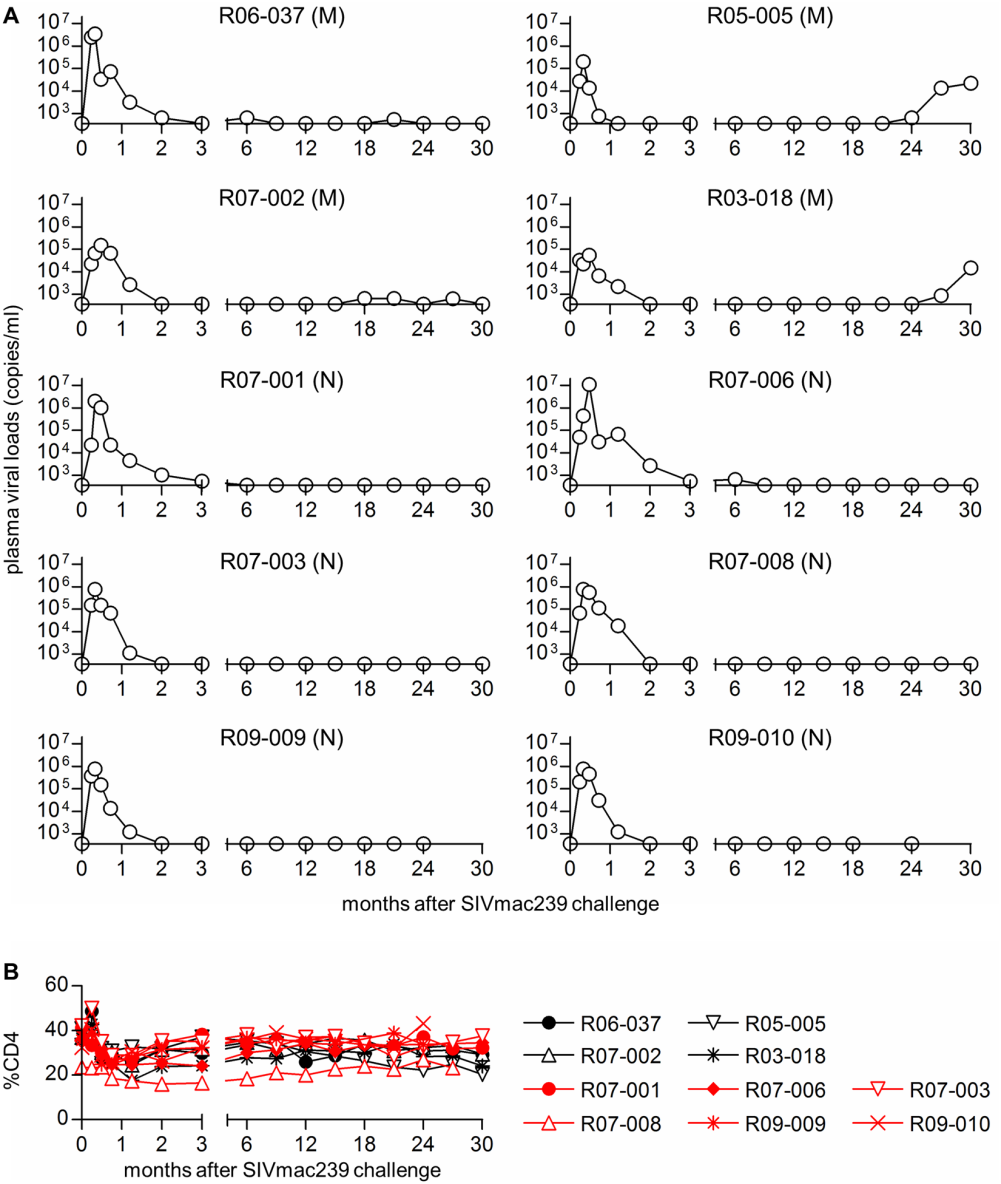
Plasma viral loads and peripheral %CD4 in SIV controllers. (A) Plasma viral loads (SIV *gag* RNA copies/ml plasma) determined as described previously [26]. The lower limit of detection is approximately 4 x 10^2^ copies/ml. On the basis of the data on proviral *gag* nucleotide sequences at 2 years post-infection, animals were divided into two groups, Group M (M) with multiple CD8^+^ T-cell escape mutations and Group N (N) with no mutation. (B) Percentage of CD4^+^ T cells in PBMCs.

Ten Burmese rhesus macaques possessing the MHC class I haplotype *90-120-Ia* that controlled viremia for 2 years after SIVmac239 challenge were studied. In our previous study [34], most A^+^ animals mounted Gag-specific and Nef-specific CD8^+^ T-cell responses in primary SIVmac239 infection. Seven *90-120-Ia*-associated CD8^+^ T-cell epitopes, Gag_206–216_, Gag_241–249_, Gag_367–381_, Nef_9–19_, Nef_89–97_, Nef_193–203_, and Vif_114–124_, in SIVmac239 antigens have been identified, and unvaccinated A^+^ animals that failed to control viral replication have been shown to select viral escape mutations from all of these epitope-specific CD8^+^ T cells within a year after SIVmac239 challenge [25,33,34]. Ten SIV controllers used in the present study consisted of two unvaccinated, one sham-vaccinated, and seven vaccinated A^+^ animals as shown in Table 1. Three macaques (R07-002, R07-003, and R07-008) were immunized with a single Gag_241–249_-epitope vaccine expressing the Gag_241–249_ epitope fused with enhanced green fluorescent protein (EGFP) [35], three macaques (R03-018, R09-009, and R09-010) with a single Gag_206–216_-epitope vaccine expressing the Gag_206–216_ epitope fused with EGFP [36], and macaque R05-005 with a mixture of both. In these vaccinated animals, plasma viremia became undetectable at 2 months post-infection, whereas unvaccinated controllers showed undetectable viremia after 6 months (Fig 1A). All the animals controlled SIV replication until 2 years, but two of them (R05-005 and R03-018) clearly exhibited reappearance of plasma viremia after that (Fig 1A). Viremia was undetectable at 1 year post-infection in all the ten SIV controllers even by quantitation of viral loads using fivefold-concentrated plasma (Table 2). However, this higher-sensitive assay detected marginal levels of viremia at 2 years post-infection in macaques R05-005 and R03-018 (Table 2). Peripheral CD4^+^ T lymphocytes were maintained in all of these SIV controllers (Fig 1B).

**Table 1:** SIV controllers analyzed in this study

Macaques	Vaccination^a^	Virus recovery^b^
Group M^c^		
R06-037	unvaccinated	**positive**
R05-005	Gag_206–216/241–249_	**positive**
R07-002	Gag_241–249_	**positive**
R03-018	Gag_206–216_	negative
Group N^c^		
R07-001	unvaccinated	negative
R07-006	control	**positive**
R07-003	Gag_241–249_	negative
R07-008	Gag_241–249_	negative
R09-009	Gag_206–216_	negative
R09-010	Gag_206–216_	negative

^a^Animals were unvaccinated or vaccinated with DNA-prime/SeV-boost expressing EGFP (control), Gag_202–216_-EGFP and Gag_236–251_-EGFP (Gag_206–216/241–249_), Gag_236–251_-EGFP (Gag_241–249_), or Gag_202–216_-EGFP (Gag_206–216_) before SIVmac239 challenge. All of them controlled SIV replication for more than 2 years.

^b^positive, viral *gag* fragments were detected by RT-PCR amplification from culture supernatant-derived RNAs of PBMCs at 2 years; negative, those were undetectable.

^c^On the basis of the data on proviral *gag* nucleotide sequences at 2 years post-infection, animals were divided into two groups, Group M with multiple mutations and Group N with no mutation.

**Table 2:** Plasma viral loads determined by using fivefold-concentrated plasma^a^

Macaques	VL at 1 year pi	VL at 2 years pi
R06-037	undetectable	ND
R05-005	undetectable	3.3 x 10^2^ copies/ml
R07-002	undetectable	undetectable
R03-018	undetectable	1.3 x 10^2^ copies/ml
R07-001	undetectable	undetectable
R07-006	undetectable	ND
R07-003	undetectable	undetectable
R07-008	undetectable	undetectable
R09-009	undetectable	undetectable
R09-010	undetectable	undetectable

^a^After centrifugation of 1 ml of plasma at 25,000 x *g* for 2 hours, 0.8 ml of its supernatant was discarded for fivefold concentration of plasma. The remaining 0.2 ml was subjected to RNA extraction for quantitation of viral loads (VL) [25]. Plasma samples of R06-037 and R07-006 at 2 years post-infection (pi) were unavailable (ND, not determined).

**Fig 2:**
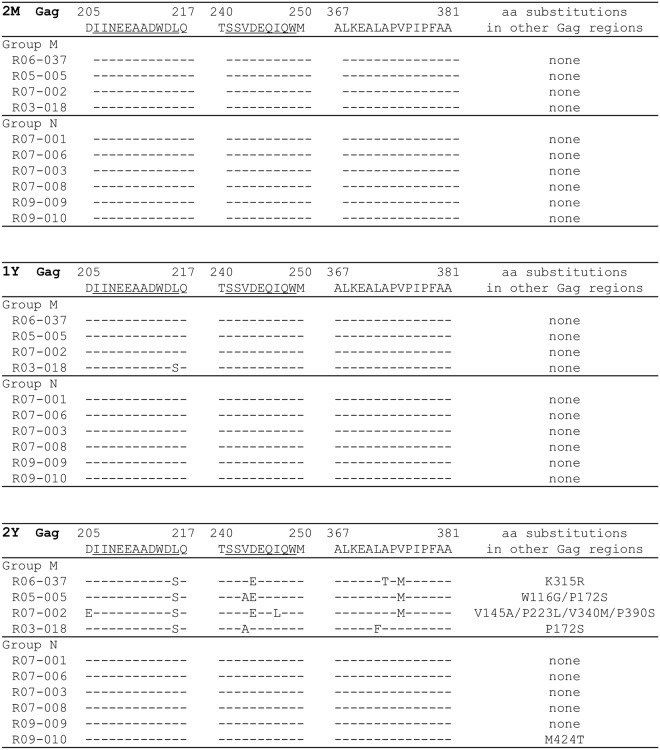
Dominant non-synonymous mutations in proviral *gag* in SIV controllers. Amino acid substitutions around SIV Gag_206–216_, Gag_241–249_, and Gag_367–381_ epitopes and in other Gag regions approximately 2 months (2M, top), 1 year (1Y, middle), and 2 years (2Y, bottom) after SIVmac239 challenge are shown. Most of the proviral gag fragments were amplified from CD4^+^ T cells isolated from PBMCs, while those at 2 years in macaques R06-037, R05-005, R07-001, and R07-006 were from cultured CD4^+^ T cells due to limitation of available cell numbers. Mutant sequences shown were completely dominant (i.e., wild-type sequences were undetectable at the residues showing mutant sequences) except for the L216S mutation (the ratio of wild type/mutant: 2/5) in macaque R03-018 at 1 year post-infection. No subdominant mutation was detected.

We examined whether viral *gag* CD8^+^ T-cell escape mutations accumulated during SIV control. Plasma viral loads were too low for us to recover viral *gag* cDNA fragments by reverse transcription (RT) and nested PCR amplification from concentrated plasma-derived RNA of these SIV controllers during the chronic phase of infection. We, therefore, analyzed sequences of proviral *gag* cDNA fragments amplified by nested PCR. Template DNAs were extracted from CD4^+^ T cells isolated from macaque peripheral blood mononuclear cells (PBMCs) obtained at approximately 2 years post-infection (Fig 2). Four animals (referred to as Group M [Table 1]) had multiple proviral *gag* mutations, exhibiting the accumulation of CD8^+^ T-cell escape mutations in the chronic phase. All Group M animals showed selection of mutations in Gag_206–216_, Gag_241–249_, and Gag_367–381_ epitope-coding regions with additional *gag* mutations. We have previously reported that the L216S (leading to a L-to-S substitution at the 216th amino acid [aa] in Gag) and D205E (D-to-E at the 205th) mutations result in escape from Gag_206–216_-specific CD8^+^ T-cell recognition [25,37]. We have also confirmed that D244E (D-to-E at the 244th) results in escape from Gag_241–249_-specific CD8^+^ T cells and that A373T (A-to-T at the 373rd) and V375M (V-to-M at the 375th) from Gag_367–381_-specific CD8^+^ T cells [25]. In contrast, the remaining six SIV controllers (referred to as Group N [Table 1]) showed no proviral mutations in Gag_206–216_, Gag_241–249_, and Gag_367–381_ epitope-coding regions. No mutation in other *gag* region was found in Group N animals except for macaque R09-010 having a single M424T mutation. Viral *gag* cDNA fragments were amplified from culture supernatants of CD4^+^ T cells derived from three of four Group M animals but not from Group N animals except for macaque R07-006 (Table 1), implying inefficient transcription or replication capacity of the proviruses with no *gag* mutations in Group N animals.

We also analyzed proviral *gag* sequences in PBMCs obtained at 2 months and 1 year after SIVmac239 challenge (Fig 2). In contrast to the results obtained at 2 years post-infection, no proviral *gag* mutations were observed in Group M or Group N animals, except macaque R03-018 (Group M) having a single L216S mutation at 1 year. Thus, Group M macaques accumulated proviruses with multiple CD8^+^ T-cell escape mutations later than 1 year post-infection and had replication-competent viruses that could be recovered from PBMC culture at 2 years.

**Fig 3:**
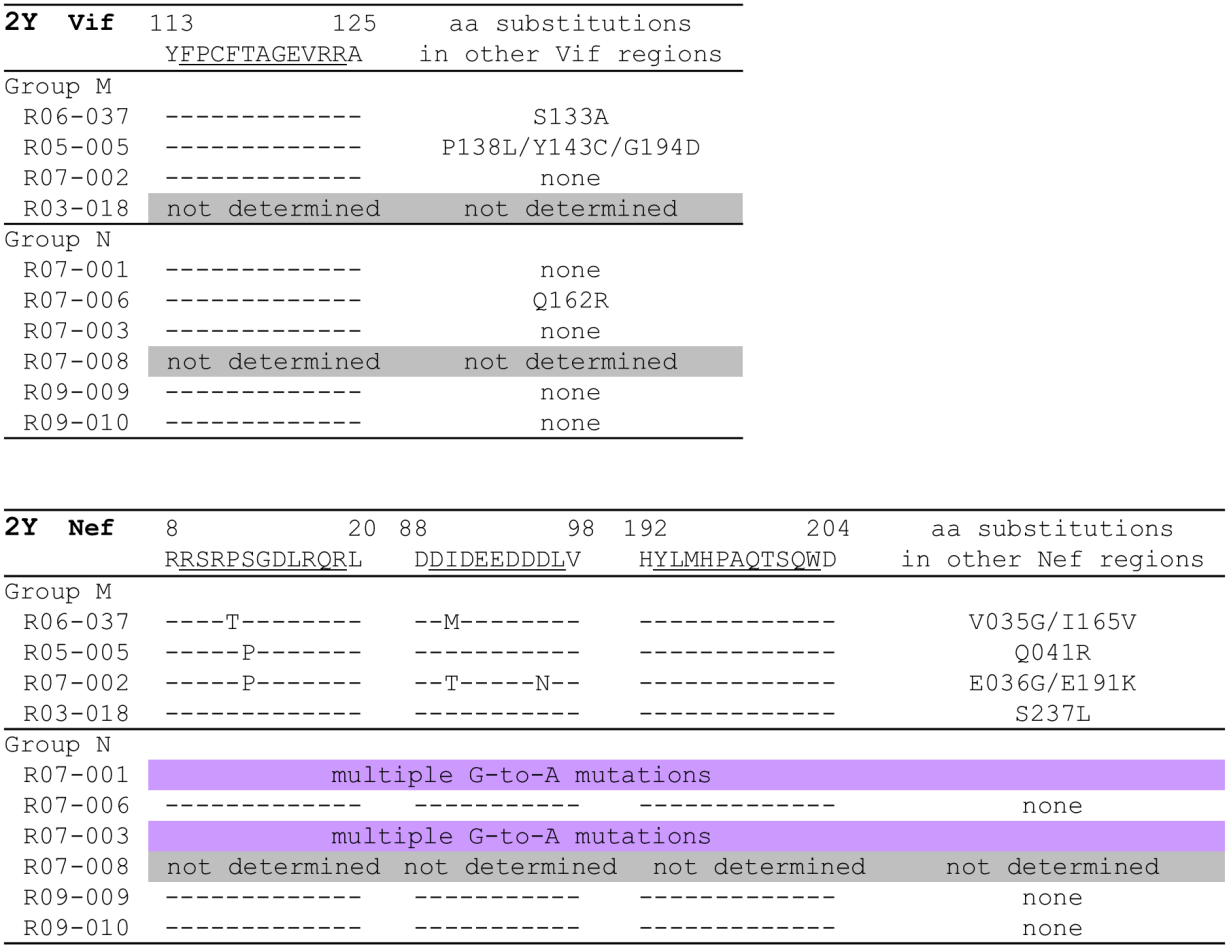
Dominant non-synonymous mutations in proviral *vif* and *nef* in SIV controllers. In the upper panel, amino acid substitutions around SIV Vif_114–124_ epitope and in other Vif regions approximately 2 years after SIVmac239 challenge are shown. In the lower panel, amino acid substitutions around SIV Nef_9–19_, Nef_89–97_, and Nef_193–203_ epitopes and in other Nef regions approximately 2 years after SIVmac239 challenge are shown. Sequences of *vif* in macaques R03-018 and R07-008 and *nef* in macaques R07-008 were not determined because these cDNA fragments could not be amplified. Macaques R07-001 and R07-003 had multiple G-to-A mutations in *nef* (See S1 Fig). Mutant sequences shown were completely dominant except for *vif* P138L (the ratio of wild type/mutant: 1/2) in R05-005, *vif* Q162R (1/1) in R07-006, *nef* P012T (1/10) in R06-037, and *nef* S013P (3/10), I090T (1/5), D096N (1/5), and E191K (1/5) in R07-002. In addition, subdominant *nef* mutations resulting in P012S (5/2) and G044E (5/2) were detected in macaque R07-002, while the wild-type sequences were dominant at these positions.

We then examined proviral *vif* and *nef* sequences at 2 years in these SIV controllers (Fig 3). Proviral *vif* cDNA fragments were amplified from three of four Group M and five of six Group N animals. Proviral *vif* mutations were found in two of the three Group M but only one of the five Group N. Neither Group M nor N had mutations in the Vif_114–124_ CD8^+^ T-cell epitope-coding region.

Group M animals had multiple proviral *nef* mutations, exhibiting accumulation of CD8^+^ T-cell escape mutations during the chronic phase of infection. Mutations in the Nef_9–19_- and Nef_89–97_-coding regions were selected in three and two of four Group M animals, respectively. We have previously reported that the P12T (P-to-T at the 12th aa in Nef) and S13P (S-to-P at the 13th) were Nef_9–19_-specific CD8^+^ T-cell escape mutations [34]. In the Nef_193–203_-coding region, however, no mutation was observed even in Group M animals. In contrast, Group N controllers showed no mutation in proviral Nef_9–19_- Nef_89–97_- and Nef_193–203_-coding regions, while macaques R07-001 and R07-003 had multiple G-to-A mutations (S1 Fig), possibly due to the effect of the APOBEC3 family [38–41]. Thus, we found two groups of A^+^ SIV controllers, Group M accumulating CD8^+^ T-cell escape mutations and Group N having no dominant CD8^+^ T-cell escape mutations in proviruses.

### SIV antigen-specific CD8^+^ T-cell responses in SIV controllers

**Fig 4:**
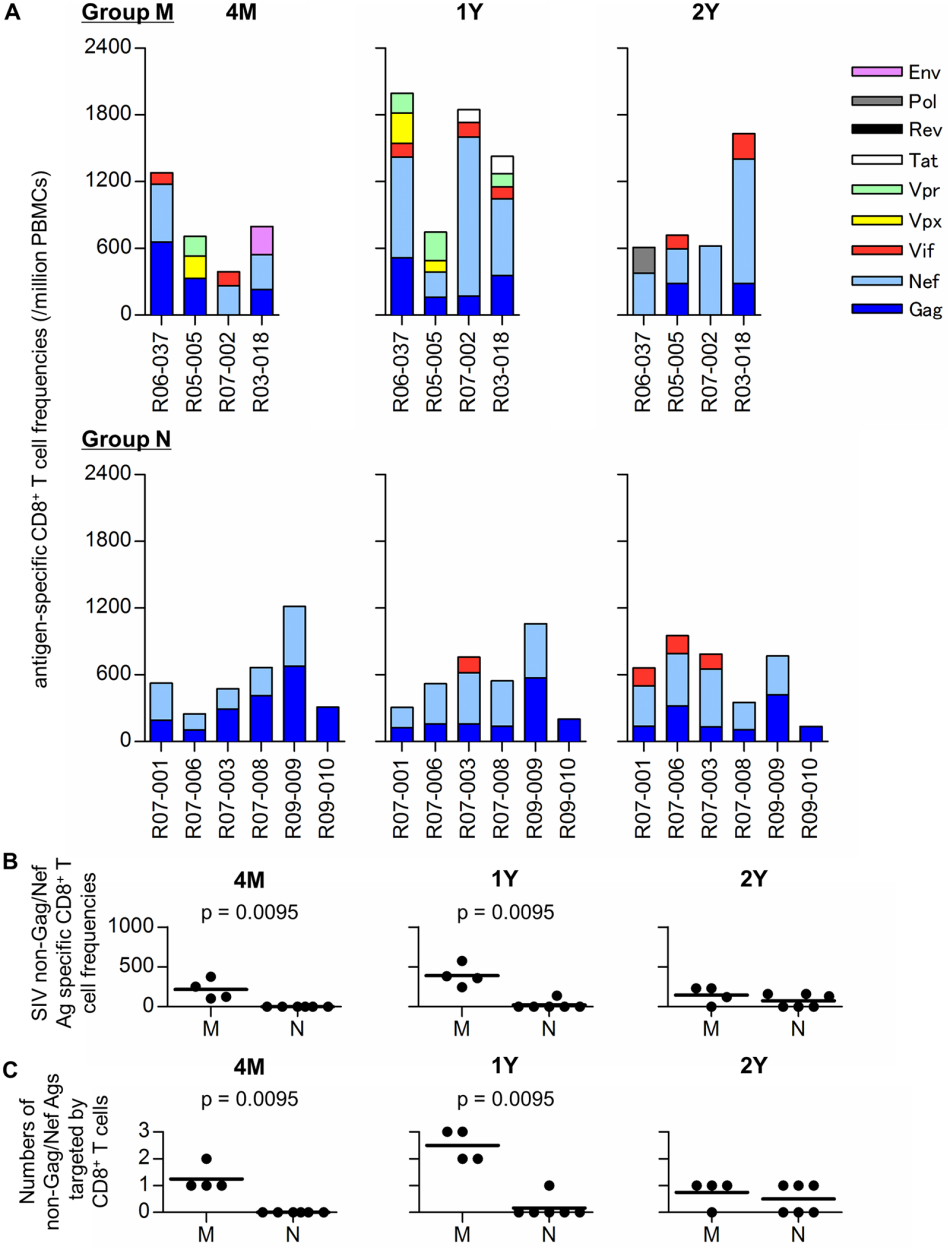
SIV antigen-specific CD8^+^ T-cell responses in SIV controllers. (A) Frequencies of CD8^+^ T cells specific for Gag, Nef, Vif, Vpx, Vpr, Tat, Rev, Pol, and Env in Group M (upper panels) and Group N (lower) at 4 months (4M), 1 year (1Y), and 2 years (2Y) post-infection. Responses were measured by detection of antigen-specific IFN-γ induction using panels of overlapping peptides spanning the entire SIVmac239 Gag, Nef, Vif, Vpx, Vpr, Tat, Rev, Pol, and Env amino acid sequences, respectively. (B) Comparisons of CD8^+^ T-cell frequencies specific for SIV antigens other than Gag and Nef at 4M, 1Y, and 2Y between Groups M and N. Group M had significantly higher frequencies of SIV non-Gag/Nef antigen-specific CD8^+^ T cells at 4M (p = 0.0095 by Mann-Whitney U-test) and 1Y (p = 0.0095). (C) Comparisons of the numbers of CD8^+^ T cell-targeted SIV antigens other than Gag and Nef at 4M, 1Y, and 2Y between Groups M and N. The numbers were significantly higher in Group M at 4M (p = 0.0095 by Mann-Whitney U-test) and 1Y (p = 0.0095).

Next, we examined individual SIV antigen-specific CD8^+^ T-cell responses in our SIV controllers by using panels of overlapping peptides spanning the entire SIVmac239 Gag, Pol, Vif, Vpx, Vpr, Tat, Rev, Env, and Nef amino acid sequences (Fig 4A). No significant difference in SIV whole antigen-specific CD8^+^ T-cell frequencies (sum of individual antigen specific CD8^+^ T-cell frequencies) was observed between Groups M and N at 4 months post-infection. However, the frequencies in Group M were significantly higher than in Group N at 1 year post-infection (p = 0.0381 by Mann-Whitney U-test). All the Group M animals had higher frequencies at 1 year than at 4 months, but Group N showed no or minimal increase in the whole antigen-specific CD8^+^ T-cell frequencies. In particular, three Group N controllers, R07-008, R09-009, and R09-010, exhibited gradual decreases in the whole antigen-specific CD8^+^ T-cell frequencies from 4 months to 2 years following challenge (Fig 4A).

All the A^+^ SIV controllers induced predominant Gag- and/or Nef-specific CD8^+^ T-cell responses at 4 months after SIVmac239 infection (Fig 4A). Group M animals elicited additional CD8^+^ T-cell responses directed against SIV antigens other than Gag and Nef. These SIV non-Gag/Nef antigen-specific CD8^+^ T-cell frequencies were significantly higher in Group M than those in Group N at 4 months (p = 0.0095 by Mann-Whitney U-test) and 1 year (p = 0.0095) (Fig 4B). Indeed, the numbers of SIV non-Gag/Nef antigens targeted by CD8^+^ T cells were significantly higher in Group M than those in Group N at 4 months (p = 0.0095 by Mann-Whitney U-test) and 1 year (p = 0.0095) (Fig 4C). All the Group M animals showed increase in the numbers of SIV antigens targeted by CD8^+^ T cells at 1 year compared to those at 4 months. In particular, SIV non-Gag antigen-specific CD8^+^ T-cell frequencies were significantly higher in Group M at 1 year (p = 0.0087). These results indicate the broadening of the CD8^+^ T-cell response in Group M.

All four animals in Group M mounted CD8^+^ T-cell responses specific for SIV non-Gag/Nef antigens, which were undetectable in all Group N animals at 4 months post-infection. Also at 1 year, all of the animals in Group M showed CD8^+^ T-cell responses specific for several targets besides the Gag and Nef antigens. In contrast, only Gag-specific and Nef-specific CD8^+^ T-cell responses were observed in Group N animals except for macaque R07-003 which had detectable Vif-specific CD8^+^ T-cell responses. At 2 years post-infection, Vif-specific CD8^+^ T-cell responses were detected in three macaques in Group N, while the remaining three showed Gag-specific and Nef-specific CD8^+^ T-cell responses only. Importantly, the latter three macaques (R07-008, R09-009, and R09-010) exhibited gradual decreases in these Gag/Nef-specific CD8^+^ T-cell responses in the chronic phase, possibly reflecting the absence of measurable viral replication.

### SIV epitope-specific CD8^+^ T-cell responses in SIV controllers

**Fig 5:**
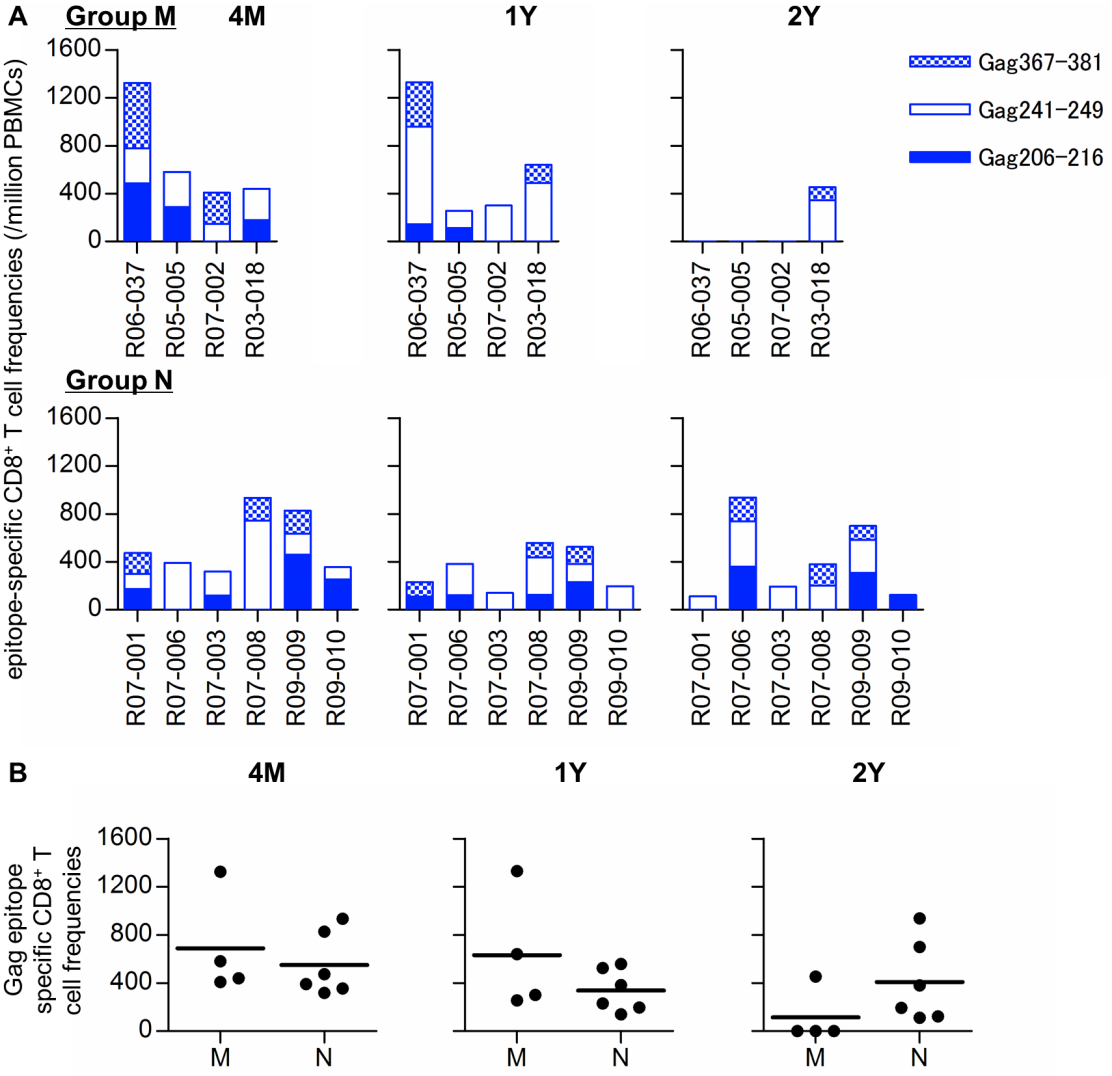
Gag_206–216_, Gag_241–249_, and Gag_367–381_ epitope-specific CD8^+^ T-cell responses in SIV controllers. (A) Frequencies of CD8^+^ T cells specific for SIV Gag_206–216_, Gag_241–249_, and Gag_367–381_ epitopes in Group M (upper panels) and Group N (lower) at 4 months (4M), 1 year (1Y), and 2 years (2Y) post-infection. (B) Comparisons of the sum of Gag_206–216_-, Gag_241–249_-, and Gag_367–381_-specific CD8^+^ T-cell frequencies at 4M, 1Y, and 2Y between Groups M and N. No significant difference was observed between the groups.

We further examined CD8^+^ T-cell responses specific for *90-120-Ia*-associated Gag epitopes; Gag_206–216_, Gag_241–249_, and Gag_367–381_, in the ten SIV controllers (Fig 5A). The sum of these Gag epitope-specific CD8^+^ T-cell frequencies was similar between Groups M and N (Fig 5B). Induction of CD8^+^ T-cell responses directed against both dominant Gag_206–216_ and Gag_241–249_ epitopes were confirmed in all the animals in previous analyses at weeks 2 and 12 post-infection [35,36]. At 4 months, all had detectable Gag_241–249_-specific CD8^+^ T cells, although Gag_206–216_-specific CD8^+^ T-cell responses were undetectable in three animals. However, at 2 years post-infection, the Gag_241–249_-specific CD8^+^ T-cell responses became undetectable in three of four Group M animals but were maintained in Group N animals except for macaque R09-010 which exhibited Gag_206–216_-specific CD8^+^ T-cell responses.

**Fig 6:**
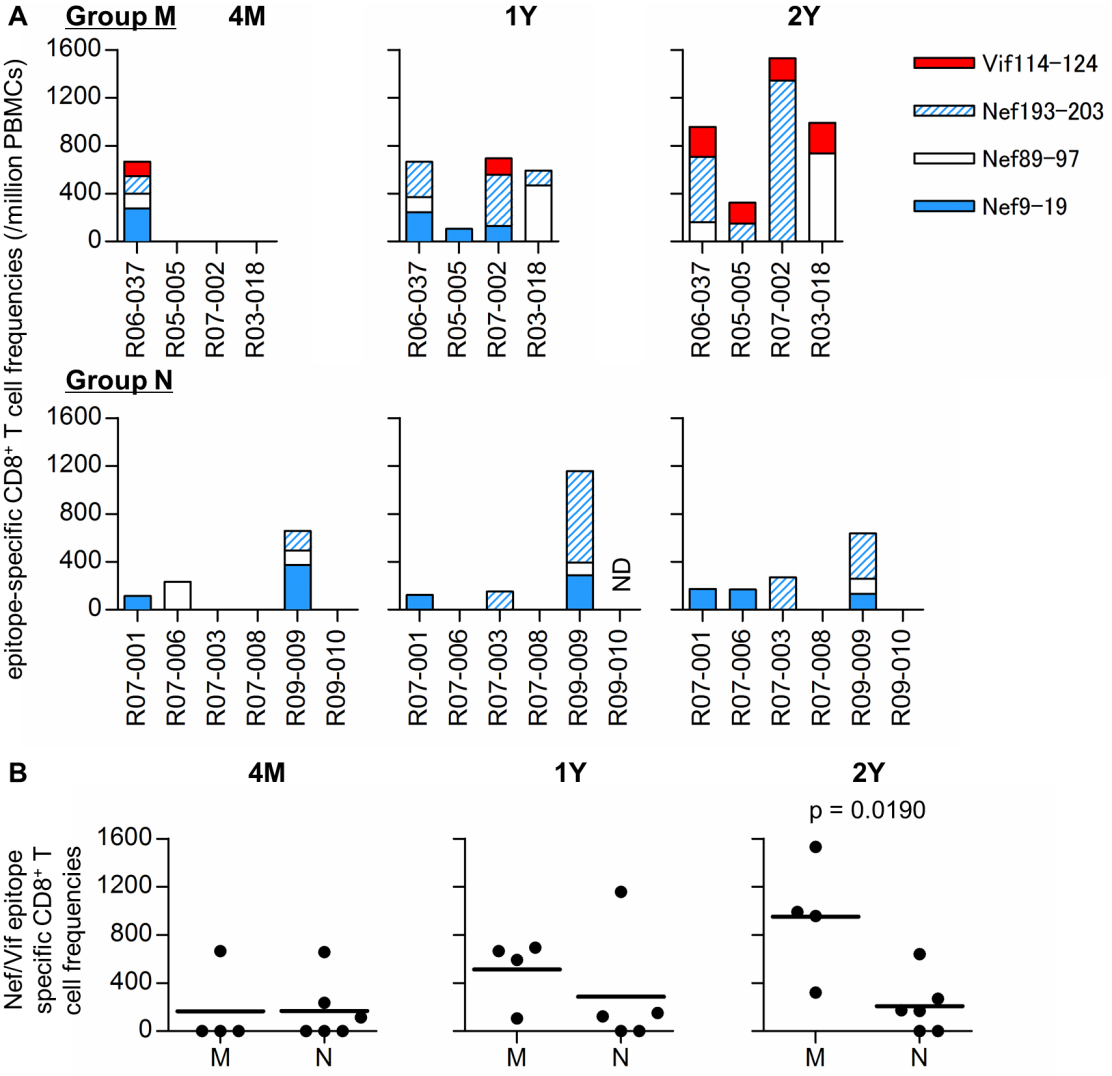
Nef_9–19_, Nef_89–97_, Nef_193–203_, and Vif_114–124_ epitope-specific CD8^+^ T-cell responses in SIV controllers. (A) Frequencies of CD8^+^ T cells specific for SIV Nef_9–19_, Nef_89–97_, Nef_193–203_, and Vif_114–124_ epitopes in Group M (upper panels) and Group N (lower) at 4 months (4M), 1 year (1Y), and 2 years (2Y) post-infection. (B) Comparisons of the sum of Nef_9–19_-, Nef_89–97_-, Nef_193–203_-, and Vif_114–124_-specific CD8^+^ T-cell frequencies at 4M, 1Y, and 2Y between Groups M and N. The sum of CD8^+^ T-cell frequencies specific for these epitopes in Group M was significantly higher compared to Group N at 2Y post-infection (p = 0.0190 by Mann-Whitney U-test).

We also examined CD8^+^ T-cell responses specific for *90-120-Ia*-associated Nef_9–19_, Nef_89–97_, Nef_193–203_, and Vif_114–124_ epitopes in the SIV controllers (Fig 6A). In most of the animals immunized with Gag_241–249_-epitope and/or Gag_206–216_-epitope vaccines (except for macaque R09-009), these Gag epitope-specific CD8^+^ T-cell responses were predominant but Nef/Vif epitope-specific CD8^+^ T-cell responses were undetectable at 4 months post-challenge. At 1 and 2 years post-infection, differences in these Nef/Vif epitope-specific CD8^+^ T-cell responses between Groups M and N became evident. All the Group M animals mounted Nef_9–19_- Nef_89–97_-, or Nef_193–203_-specific CD8^+^ T-cell responses at 1 year and Vif_114–124_-specific CD8^+^ T-cell responses at 2 years post-infection. In contrast, none of the Group N animals elicited Vif_114–124_-specific CD8^+^ T-cell responses. In two of them, macaques R07-008 and R09-010, even Nef_9–19_- Nef_89–97_-, and Nef_193–203_-specific CD8^+^ T cells were undetectable. Indeed, the sum of Nef_9–19_- Nef_89–97_-, Nef_193–203_-, and Vif_114–124_-specific CD8^+^ T-cell frequencies in Group M was significantly higher than in Group N at 2 years (p = 0.0190 by Mann-Whitney U-test) (Fig 6B). Thus, Group M animals mounted Nef and Vif epitope-specific CD8^+^ T-cell responses in the chronic phase of infection, whereas such broadening of CD8^+^ T-cell responses was unclear and Gag epitope-specific CD8^+^ T cells were maintained in Group N. In particular, in three of six Group N macaques, R07-008, R09-009, and R09-010, comparison between 4 months and 2 years post-challenge showed no change in CD8^+^ T-cell target epitopes.

### CD8^+^ cell-depletion in a Group N controller

**Fig 7:**
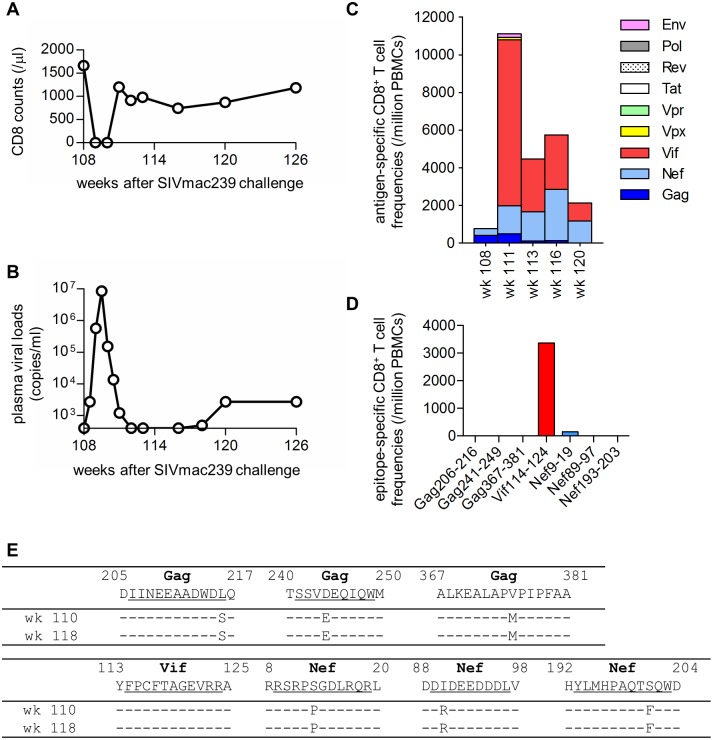
Virological and immunological analyses in macaque R09-009 following CD8^+^ cell depletion. (A) Changes in peripheral CD8^+^ T-cell counts after the initial anti-CD8 antibody administration. Group N macaque, R09-009, was administered anti-CD8 antibody at week 108 post-infection and on days 3, 7, and 10 after the first administration. (B) Changes in plasma viral loads. (C) Changes in CD8^+^ T-cell responses specific for SIV Gag, Nef, Vif, Vpx, Vpr, Tat, Rev, Pol, and Env. (D) CD8^+^ T-cell responses specific for SIV Gag_206–216_, Gag_241–249_, Gag_367–381_, Vif_114–124_, Nef_9–19_, Nef_89–97_, and Nef_193–203_ epitopes at week 113 post-infection. (E) Dominant non-synonymous mutations in plasma viral cDNA regions encoding Gag, Vif, and Nef epitopes. Viral *gag*, *vif*, and *nef* cDNA fragments were amplified from plasma RNA obtained at weeks 110 and 118 post-infection. Amino acid substitutions around SIV Gag_206–216_, Gag_241–249_, Gag_367–381_, Vif_114–124_, Nef_9–19_, Nef_89–97_, and Nef_193–203_ epitopes are shown.

Three of six Group N controllers, R07-008, R09-009, and R09-010, showed gradual decreases and stable breadth in the SIV antigen-specific CD8^+^ T-cell responses from 4 months to 2 years post-infection. To confirm involvement of the CD8^+^ T-cell responses in this stable SIV control, we administered an anti-CD8 antibody to deplete the CD8^+^ cells in macaque R09-009 2 years following challenge. Peripheral CD8^+^ T cells were undetectable for approximately 2 weeks from day 3 after the initial anti-CD8 antibody administration at week 108 post-infection and became detectable at week 111 (Fig 7A). Plasma viremia was detectable for approximately 3 weeks from day 3 after the initial anti-CD8 antibody administration and became undetectable at week 112 (Fig 7B). This transient reappearance of plasma viremia concomitant with CD8^+^ cell depletion supports the notion that CD8^+^ T-cell responses are crucial for the stable SIV containment.

Macaque R09-009 showed Gag- and Nef-specific but not non-Gag/Nef antigen-specific CD8^+^ T-cell responses before the anti-CD8 antibody administration at week 108 post-infection (Fig 4A). Interestingly, however, this animal mounted high magnitude Vif-specific CD8^+^ T-cell responses in the CD8^+^ T-cell recovery phase from week 111 post-infection (Fig 7C). Analysis at week 115 detected no or poor Gag/Nef epitope-specific CD8^+^ T cells but dominant Vif_114–124_ epitope-specific CD8^+^ T-cell responses (Fig 7D). The plasma-derived viral genome cDNAs at week 110 post-infection had mutations L216S in the Gag_206–216_ epitope-coding region, D244E in Gag_241–249_, V375M in Gag_367–381_, S13P in Nef_9–19_, I90R in Nef_89–97_, and S201F in Nef_193–203_ (Fig 7E). However, no mutation was detected in the Vif_114–124_ epitope-coding region. Plasma viremia re-appeared at week 118, and the viruses at week 118 showed the similar pattern of sequences (Fig 7E). Thus, even in lasting aviremic SIV controllers, replication-competent viruses with multiple CD8^+^ T-cell escape mutations may exist.

## Discussion

In the present study, we examined ten rhesus macaques that controlled SIV replication without detectable viremia for more than 2 years after infection. Four of these aviremic SIV controllers, Group M, exhibited proviruses with multiple proviral CD8^+^ T-cell escape mutations in the chronic phase, whereas the remaining six, Group N, had no proviral CD8^+^ T-cell escape mutations. Three of six Group N animals showed a decline in SIV-specific CD8^+^ T-cell frequencies, implying lasting non-sterile SIV control with a concomitant reduction in viral replication. Although the size and character of virus reservoirs may be different from those under ART [42,43], a rhesus cytomegalovirus (rhCMV) vector vaccine trial indicated lasting, non-sterile SIV control by persistent rhCMV-induced CD8^+^ T-cell responses, possibly resulting in virus clearance [44]. Our results suggest possible achievement of lasting SIV control by CD8^+^ T cells without persistent exogenous stimulation. We were unable to find the genetic determinants for the difference in the Groups M and N by analyses of their second MHC class I haplotypes (Table 3), but this study presents a model of SIV containment, contributing to elucidation of the requisites for lasting, non-sterile HIV control.

**Table 3:** Alleles in the second MHC-I haplotypes in macaques^a^

Macaques	Alleles
R06-037	A1*052:01, A2*05:13, B*089:02/03
R05-005	A1*105:02, B*056:03, B*066:01
R07-002	A1*066:01, B*021:02
R03-018	A1*018:08, A2*05:31, B*001:01, B*007:02/03
R07-001	A1*032:02, B*066:01
R07-006	A1*032:02
R07-003	A1*066:01, B*003:01, B*005:01, B*007:02/03, B*015:04
R07-008	A1*004:01, A1*018:01, A2*05:31, B*043:01
R09-009	A1*107:01, B*046:03
R09-010	B*063:02, B*066:01

^a^Detected alleles not included in the first MHC-I haplotype *90-120-Ia* are shown.

This study analyzed SIV controllers possessing the MHC-I haplotype *90-120-Ia*. In our previous study [31], all the A^+^ animals vaccinated with a DNA prime and an SeV-Gag boost controlled replication of the wild-type SIVmac239, whereas those that received the same vaccine regimen failed to control a challenge with SIV carrying Gag_206–216_- and Gag_241–249_-specific CD8^+^ T-cell escape mutations. Thus, these A^+^ animals are useful to examine the mechanism of SIV control by CD8^+^ T cells, and the present study using these SIV controllers may provide a clue to understand the mechanism for lasting SIV control. Analysis of CD8^+^ T cell-mediated SIV control needs animals sharing MHC-I genotypes like A^+^ macaques. If we could accumulate long-lasting aviremic SIV controllers sharing other MHC-I alleles, although not easy, it would contribute to our further understanding of viral control mechanism.

Gag_206–216_- and Gag_241–249_-specific CD8^+^ T-cell responses played a central role in primary viral control in these A^+^ SIV controllers as shown previously [31, 35,36]. In most vaccine-based controllers, a viral CD8^+^ T-cell escape GagL216S mutation was rapidly selected and plasma viremia became undetectable after 5 weeks of infection. However, the wild-type sequence but not this CD8^+^ T-cell escape mutation was predominant in PBMC-derived proviruses at 2 months post-infection, reflecting containment of the rapidly-selected mutant viruses. The data infer that the detected proviruses with wild-type *gag* were maintained without eradication because of inefficient replication. Indeed, no virus was recovered from PBMC cultures in most Group N animals (Table 1). How these proviruses became non-functional remains unclear [45,46], but two Group N animals showed multiple G-to-A mutations in proviral *nef* (S1 Fig), possibly reflecting an effect of the APOBEC3 family [38–41].

Two years after infection, animals in Group M showed multiple CD8^+^ T-cell escape mutations in proviral *gag* and *nef*, suggesting replication of viruses carrying multiple mutations. In our previous study [33], all the four MHC-I haplotype *90-120-Ia*-positive macaques that failed to control SIVmac239 replication showed CD8^+^ T-cell responses targeting SIV non-Gag/Nef as well as Gag/Nef antigens at 3 months and/or 1 year post-infection. In all of these animals, analysis of plasma RNA-derived viral genome sequences at 1 year post-infection found predominant nonsynonymous mutations in all the seven regions encoding SIV Gag_206–216_, Gag_241–249_, Gag_367–381_, Vif_114–124_, Nef_9–19_, Nef_89–97_, and Nef_193–203_ epitopes, respectively. In the present study, analysis of these MHC-I haplotype *90-120-Ia*-associated epitopes at 2 years showed Gag_206–216_, Gag_241–249_, and Gag_367–381_ epitope-specific CD8^+^ T-cell escape mutations in all Group M animals. Nef_9–19_-specific CD8^+^ T-cell escape mutations were also selected in most of them. However, mutations were not observed in Nef_193–203_ or Vif_114–124_ epitope-coding region. At 2 years after infection, only one of four Group M animals maintained Gag_206–216_/Gag_241–249_-specific CD8^+^ T cells, whereas all elicited Nef_193–203_ and Vif_114–124_ epitope-specific CD8^+^ T-cell responses, implying that these CD8^+^ T cells targeting Nef_193–203_ and Vif_114–124_ epitopes may contribute to sustained control of viremia in Group M animals.

These results suggest undetectable level of viral replication in Group M animals, resulting in accumulation of CD8^+^ T-cell escape mutations during viremia control. Higher SIV-specific CD8^+^ T-cell frequencies and broadening of the CD8^+^ T-cell targets in this group are considered to reflect this undetectable level of viral replication. Proviral CD8^+^ T-cell escape mutations were undetectable at 1 year but accumulated at 2 years in Group M, while differences in SIV non-Gag/Nef antigen-specific CD8^+^ T-cell responses between Groups M and N were evident as early as 4 months post-infection. This implies that broadening of the CD8^+^ T-cell responses in aviremic SIV controllers could serve as an indicator of the beginning of viral control failure. All the Group M animals showed reduced numbers of non-Gag/Nef antigens targeted by CD8^+^ T cells at 2 years compared to those at 1 year (Fig 4). It is speculated that this might be because viruses accumulate CD8^+^ T-cell escape mutations in those viral genome regions encoding CD8^+^ T cell-targeted non-Gag/Nef antigens after 1 year post-infection.

In contrast, proviral mutations did not appear in Group N macaques even at 2 years post-infection, indicating sustained viral control. These animals maintained Gag_241–249_-specific CD8^+^ T cells which are considered important for viral control. The breadth and magnitude of SIV-specific CD8^+^ T-cell responses remained constant in the chronic phase consistent with stable viral control in the presence of immunodominant Gag- and Nef-specific CD8^+^ T-cell responses. In three Group N animals (R07-001, R07-006, and R07-003), Vif-specific CD8^+^ T-cell responses became detectable at 2 years after infection, implying partial control failure by the Gag- and Nef-specific CD8^+^ T-cell responses. However, the remaining three vaccinated controllers (R07-008, R09-009, and R09-010) in Group N showed no increased breadth of their CD8^+^ T-cell targets, possibly reflecting the absence of escape mutants stimulating additional CD8^+^ T-cell responses in the chronic phase.

Effective and broad CD8^+^ T-cell responses have been indicated to be important for the control of HIV/SIV replication [15,47–50]. Even if aviremic HIV/SIV control is achieved by virus-specific potent CD8^+^ T-cell responses, residual viral replication may occur and allow accumulation of CD8^+^ T-cell escape mutations in viral genome, possibly leading to eventual viremia rebound. The present study addressed this issue after achievement of primary SIV control. The rhCMV vector vaccine trial indicated lasting SIV control by persistent rhCMV-induced non-classical CD8^+^ T-cell responses in rhesus macaques [44,51]. However, SIV controllers in the present study are thought to have no CD8^+^ T-cell stimulators other than virus-derived antigens post-infection. Thus, broadening of CD8^+^ T-cell responses is considered to be due to residual viral replication as observed in Group M animals. Broader CD8^+^ T-cell responses would be important for SIV control, but after achievement of a virus control condition, further broadening may indicate residual viral replication. In contrast, Group N animals, in particular three of them (R07-008, R09-009, and R09-010), showed no broadening of their CD8^+^ T-cell targets, which may represent a status of lasting SIV containment by CD8^+^ T cells.

The anti-CD8 antibody administration to the Group N macaque resulted in transient reappearance of plasma viremia concomitant with CD8^+^ cell depletion, supporting the notion that CD8^+^ T-cell responses are crucial for the stable SIV control observed. Sequence analysis of the emergent virus indicated the existence of replication-competent viruses with multiple CD8^+^ T-cell escape mutations, which can be controlled.

In summary, this study showed that increased breadth of virus-specific CD8^+^ T-cell responses is detected before the accumulation of proviral CD8^+^ T-cell escape mutations and viral control failure in aviremic SIV controllers. Broadly-reactive CD8^+^ T-cell responses may be crucial for HIV control, but our results suggest that if the host could achieve the conditions in which CD8^+^ T cells overwhelm HIV replication, non-broadening of CD8^+^ T-cell responses represents a status of lasting HIV containment by CD8^+^ T cells.

## Materials and Methods

### Ethics statement

Animal experiments were carried out in Tsukuba Primate Research Center, National Institute of Biomedical Innovation (NIBP; currently renamed National Institutes of Biomedical Innovation, Health and Nutrition [NIBIOHN]) with the help of the Corporation for Production and Research of Laboratory Primates after approval by the Committee on the Ethics of Animal Experiments of NIBP (permission number: DS21-27, DS23-19, and DS25-31) under the guideline for animal experiments at NIBP and National Institute of Infectious Diseases in accordance with the Guidelines for Proper Conduct of Animal Experiments established by Science Council of Japan (http://www.scj.go.jp/ja/info/kohyo/pdf/kohyo-20-k16-2e.pdf). The experiments were in accordance with the "Weatherall report for the use of non-human primates in research" recommendations (https://royalsociety.org/topics-policy/publications/2006/weatherall-report/)). Animals were housed in adjoining individual primate cages allowing them to make sight and sound contact with one another for social interactions, where the temperature was kept at 25°C with light for 12 hours per day. Animals were fed with apples and commercial monkey diet (Type CMK-2, Clea Japan, Inc.). Blood collection, vaccination, virus challenge, and anti-CD8 antibody treatment were performed under ketamine anesthesia.

### Animal experiments

We analyzed the chronic phase of SIVmac239 infection in ten Burmese rhesus macaques (*Macaca mulatta*) possessing the MHC class I haplotype *90-120-Ia* [32,33] (Table 1). These animals were previously used for vaccination and challenge experiments [35,36]. One-third of unvaccinated A^+^ animals controlled viremia after SIVmac239 infection in our previous study [35], and unvaccinated macaques R06-037 and R07-001 and sham-vaccinated R07-006 were used in the present study. Macaque R07-006 received a control prime-boost vaccine using a DNA and a replication-incompetent F-deleted SeV (F[–]SeV) vector both expressing EGFP. Three macaques R07-002, R07-003, and R07-008 received a DNA-prime/F(-)SeV-boost vaccine eliciting Gag_241–249_-specific CD8^+^ T-cell responses. A pGag_236-250_-EGFP-N1 DNA and an F(-)SeV-Gag_236-250_-EGFP vector both expressing an SIVmac239 Gag_236-250_ (IAGTTSSVDEQIQWM)-EGFP fusion protein were used for the single Gag_241–249_-epitope vaccine [35]. Three macaques R03-018, R09-009, and R09-010 received a DNA-prime/F(-)SeV-boost vaccine eliciting Gag_206–216_-specific CD8^+^ T-cell responses. A pGag_202–216_-EGFP-N1 DNA and an F(-)SeV-Gag_202–216_-EGFP vector both expressing an SIVmac239 Gag_202–216_ (IIRDIINEEAADWDL)-EGFP fusion protein were used for the single Gag_206–216_-epitope vaccine [36]. Macaque R05-005 received both the Gag_241–249_-epitope and Gag_206–216_-epitope vaccines simultaneously. Animals received 5 mg of DNA intramuscularly and 6 weeks later received a single intranasal boost with 6 x 10^9^ cell infectious units of F(-)SeV vector. Approximately 3 months after the F(-)SeV boost, animals were challenged intravenously with 1,000 50% tissue culture infective doses of SIVmac239 [52]. For CD8^+^ cell depletion, macaque R09-009 received a single subcutaneous inoculation of 10 mg/kg of body weight of monoclonal anti-CD8 antibody (cM-T807) (NIH Nonhuman Primate Reagent Resource [R24 RR016001, N01 AI040101]) followed by three intravenous inoculations of 5 mg/kg cM-T807 on days 3, 7, and 10 after the first inoculation at week 108.

### Proviral cDNA sequencing

Primary CD4^+^ T cells were prepared by negative selection from macaque PBMCs using a non-human primate CD4^+^ T cell isolation kit (Miltenyi). Total cellular DNA was extracted from CD4^+^ T cells using DNeasy extraction kit (QIAGEN). The DNA corresponding to the number of CD4^+^ T cells indicated in S1 Table was subjected to nested PCR amplification of proviral *gag*, *vif*, and *nef* cDNA fragments (nucleotide numbers [nt] 1231–2958 for *gag*, nt 4829–7000 for *vif*, and nt 8677–10196 for *nef* in SIVmac239 [accession number M33263]) for direct sequencing using dye terminator chemistry and an automated DNA sequencer (Applied Biosystems) as previously described [25]. For macaques R06-037, R05-005, R07-001, and R07-006 at 2 years, total cellular DNAs were extracted not directly from CD4^+^ T cells but after 8 days of culture described below. Dominant non-synonymous mutations were determined. For virus recovery, 0.5–2 x 10^6^ CD4^+^ T cells were cultured in the presence of 10 ng/ml human interleukin-7 (IL-7) (Miltenyi) and 10 ng/ml human IL-15 (Miltenyi) for 8 days. Then, viral RNA was extracted from supernatants of CD4^+^ T-cell culture using the High Pure Viral RNA kit (Roche Diagnostics) and subjected to reverse transcription and nested PCR (RT-PCR) amplification of viral *gag* cDNA fragments.

### Analysis of antigen-specific CD8^+^ T-cell responses

We measured virus-specific CD8^+^ T-cell frequencies by flow cytometric analysis of gamma interferon (IFN-γ) induction after specific stimulation as described previously [53]. Autologous herpesvirus papio-immortalized B-lymphoblastoid cell lines (B-LCLs) were pulsed with individual SIVmac239 epitope-coding peptides (at a final concentration of 1–5 μM) or peptide pools (at a final concentration of 1–2 μM for each peptide) using panels of overlapping peptides spanning the entire SIVmac239 Gag, Pol, Vif, Vpx, Vpr, Tat, Rev, Env, and Nef amino acid sequences (Sigma-Aldrich Japan) for 1 hour. PBMCs were cocultured with these pulsed B-LCLs in the presence of GolgiStop (monensin, BD) for 6 hours. Intracellular IFN-γ staining was performed with a CytofixCytoperm kit (BD) and fluorescein isothiocyanate (FITC)-conjugated anti-human CD4 (BD), peridinin chlorophyll protein (PerCP)-conjugated anti-human CD8 (BD), allophycocyanin-Cy7 (APC-Cy7)-conjugated anti-human CD3 (BD), and phycoerythrin (PE)-conjugated anti-human IFN-γ monoclonal antibodies (Biolegend). In the flow cytometric analysis, PBMCs were gated in Forward Scatter-Side Scatter dot plots and B-LCLs were excluded. A representative gating schema for flow cytometric analysis is shown in S2 Fig. Specific T-cell frequencies were calculated by subtracting nonspecific IFN-γ^+^ T-cell frequencies from those after peptide-specific stimulation. Specific T-cell frequencies lower than 100 per million PBMCs were considered negative.

### Statistical analysis

Statistical analyses were performed with Prism software version 4.03 with significance levels set at a P value of <0.0500 (GraphPad Software, Inc.). Comparisons between groups were made using non-parametric tests.

### Accession numbers

SIVmac239 proviral DNA: M33263.

## Supporting Information

S1 Fig Sequences of proviral *nef* cDNAs amplified from PBMCs at 2 years after SIV challenge in macaques R07-001 and R07-003. (TIF)

S2 Fig A representative gating schema for flow cytometric analysis. (TIF)

S1 Table The number of CD4^+^ T cells used for PCR amplification of proviral *gag* cDNA fragments for sequencing. (PDF)
