# Supplementary figures and images for: Broadening of Virus-Specific CD8+ T-Cell Responses Is Indicative of Residual Viral Replication in Aviremic SIV Controllers
Source: PLoS Pathog. 2015 Nov 4;11(11):e1005247. doi: 10.1371/journal.ppat.1005247 (PMC4633064; doi:10.1371/journal.ppat.1005247)

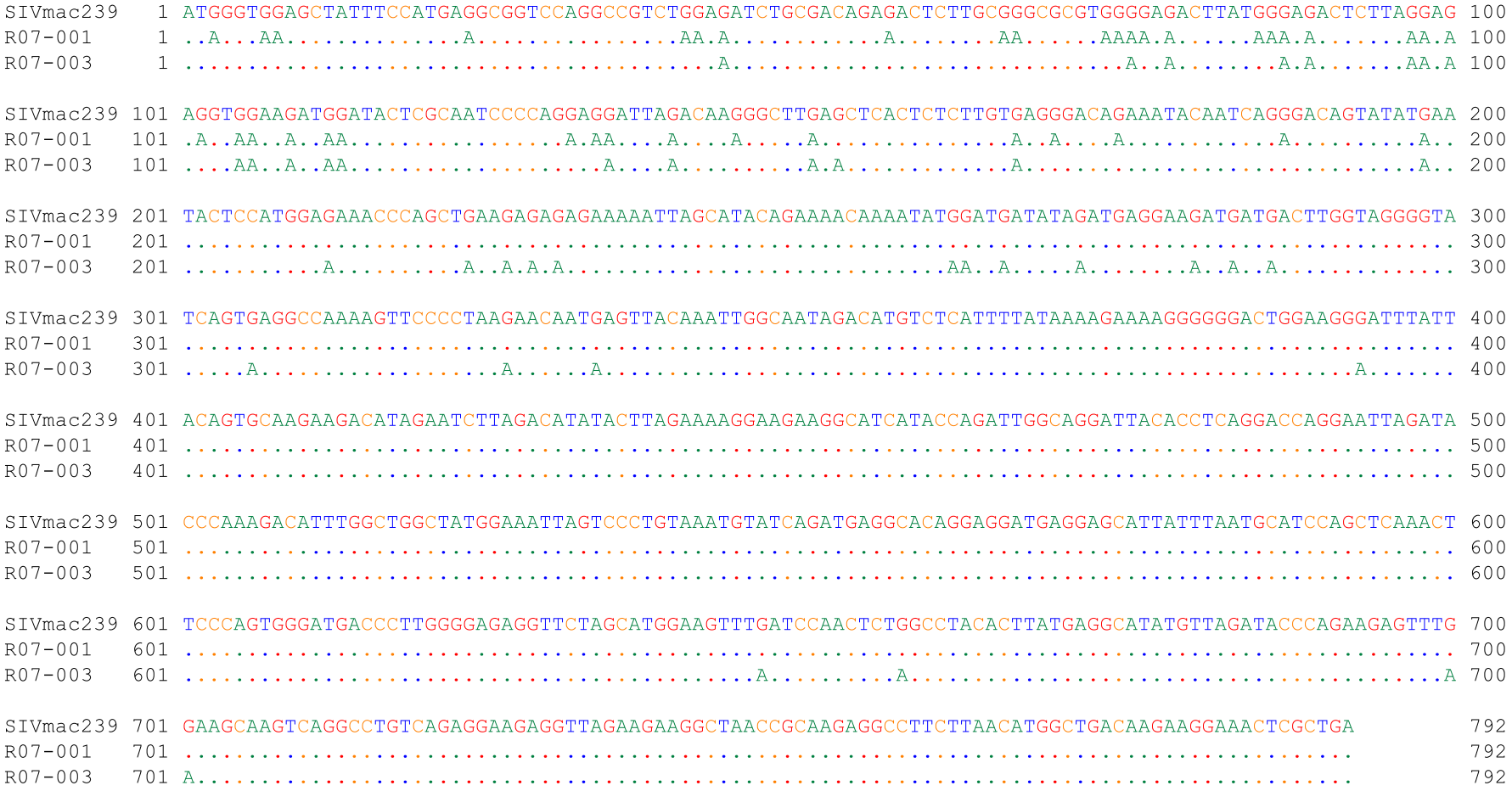

Supplement: S1 Fig — (TIF) [file ppat.1005247.s001.tif]

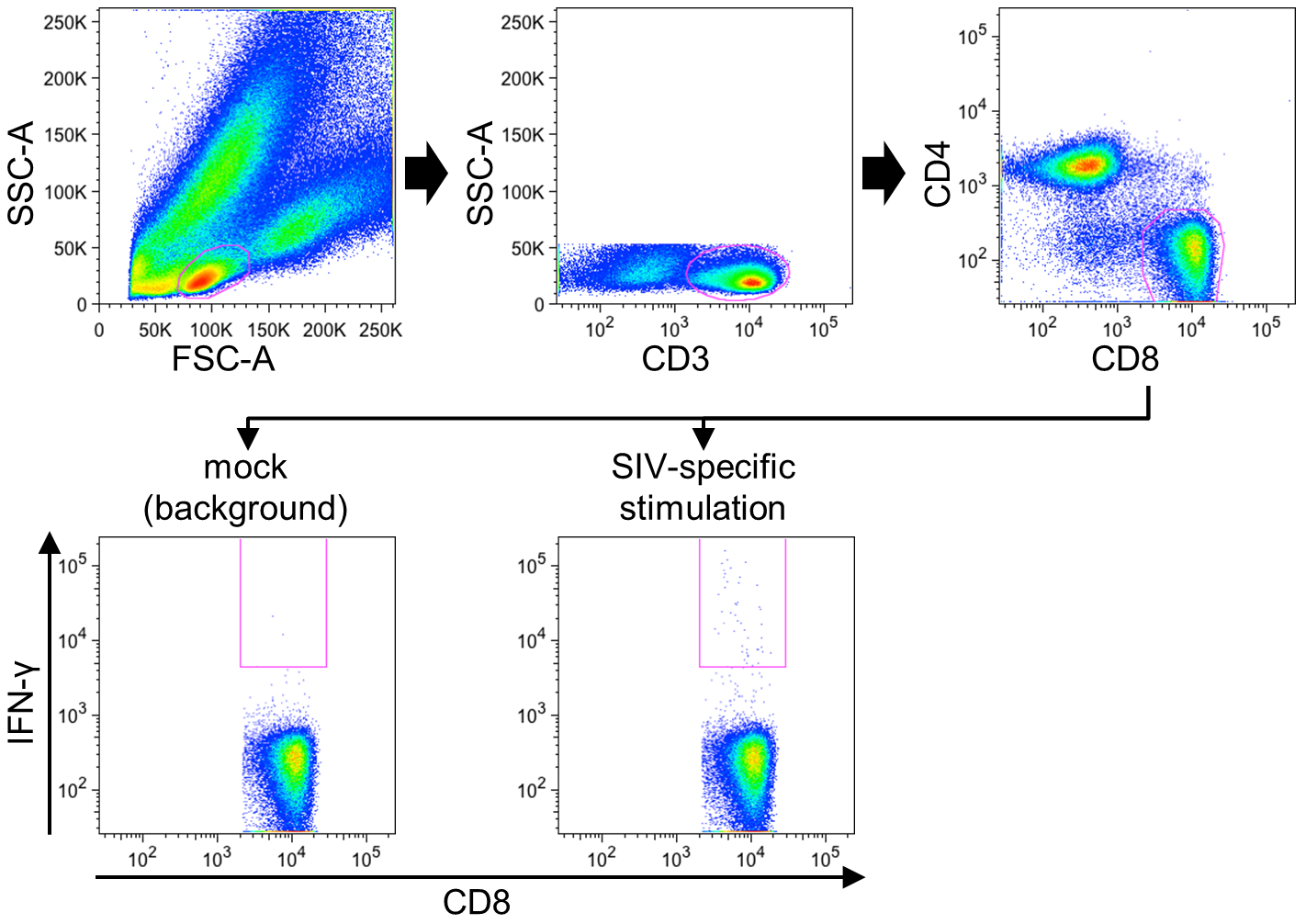

Supplement: S2 Fig — (TIF) [file ppat.1005247.s002.tif]
