# Supplementary material for: Broadening of Virus-Specific CD8+ T-Cell Responses Is Indicative of Residual Viral Replication in Aviremic SIV Controllers
Source: PLoS Pathog. 2015 Nov 4;11(11):e1005247. doi: 10.1371/journal.ppat.1005247 (PMC4633064; doi:10.1371/journal.ppat.1005247)
Supplement: S1 Table — (PDF) [file ppat.1005247.s003.pdf]

S1 Table. The number of $CD4^+$ T cells used for PCR amplification of proviral *gag* cDNA fragments for sequencing

Macaques	CD4$^+$ T-cell numbers[a]		
	2 months pi	1 year pi	2 years pi
R06-037	8.6×10^4	1.8×10^5	*6.6×10^4
R05-005	6.2×10^4	2.0×10^5	*9.4×10^4
R07-002	6.2×10^4	1.3×10^5	5.5×10^4
R03-018	5.0×10^4	1.1×10^5	4.4×10^4
R07-001	1.3×10^5	2.2×10^5	*1.0×10^5
R07-006	5.5×10^4	2.0×10^5	*1.0×10^5
R07-003	7.2×10^4	1.8×10^5	8.6×10^4
R07-008	5.5×10^4	4.4×10^5	8.1×10^4
R09-009	5.5×10^4	1.1×10^5	1.1×10^5
R09-010	6.6×10^4	1.7×10^5	6.1×10^4

[a]PBMCs obtained from macaques at 2 months, 1 year, and 2 years post-infection (pi) were used. Cellular DNAs were extracted from $CD4^+$ T cells isolated from PBMCs and were used as the template for nested PCR amplification of proviral *gag* cDNA fragments for sequencing. The amount of DNA used as the template for the first PCR amplification is corresponding to that derived from the indicated number of $CD4^+$ T cells. In macaques R06-037, R05-005, R07-001, and R07-006 at 2 years indicated by the asterisk (*), cellular DNAs were extracted after 8 days of culture. The cell numbers are different among samples because of difference in available cell numbers.
